# *Bacillus haimaensis* sp. nov.: a novel cold seep-adapted bacterium with unique biosynthetic potential

**DOI:** 10.1128/aem.02456-24

**Published:** 2025-04-25

**Authors:** Yuanyuan Wang, Luyi Yang, Wenbo Wu, Zhengqi Feng, Jian He, Changjun Guo, Jianguo He

**Affiliations:** 1 School of Marine Sciences, State Key Laboratory for Biocontrol & Southern Marine Science and Engineering Guangdong Laboratory (Zhuhai), Guangdong Province Key Laboratory of Aquatic Economic Animals / Guangdong Provincial Observation and Research Station for Marine Ranching of the Lingdingyang Bay, Sun Yat-sen University 26469 , Guangzhou, China; Colorado School of Mines, Golden, Colorado, USA

**Keywords:** *Bacillus haimaensis*, deep-sea cold seep, extremophile adaptation, biosynthetic gene clusters, comparative genomics, marine bioremediation

## Abstract

Deep-sea cold seeps harbor unique microbial communities that play crucial roles in biogeochemical cycles and possess potential biotechnological applications. Herein, we report the isolation, characterization, and genomic analysis of a novel *Bacillus* species, *Bacillus haimaensis* sp. nov. (type strain CSS-39^T^, CCTCC M20241382), obtained from sediments collected at a depth of 1,350 m in the Haima cold seep, South China Sea. Phylogenomic analysis, revealing an average nucleotide identity of 87.78% and a digital DNA-DNA hybridization value of 34.0% with its closest relative *B. tianshenii* DSM 25879^T^, confirms the taxonomic novelty of the genus *Bacillus*. The complete 4.54 Mb genome of *B. haimaensis* reveals adaptations to the cold seep environment, including enhanced nutrient acquisition capabilities and stress response mechanisms. Comparative genomic analysis identifies 27 unique gene clusters related to spore germination and sulfate assimilation, suggesting specialized metabolic strategies for this extreme habitat. Furthermore, six biosynthetic gene clusters, including a novel lassopeptide cluster, indicate a potential for secondary metabolite production. Phenotypic characterization demonstrates the strain’s ability to utilize diverse carbon sources and tolerate a wide range of environmental conditions. Our findings provide insights into microbial adaptations to deep-sea cold seeps and highlight the potential of *B. haimaensis* for biotechnological applications in bioremediation and natural product discovery. This study expands our understanding of microbial diversity in extreme marine environments and offers a new model bacterium for investigating bacterial adaptations to deep-sea ecosystems.

**IMPORTANCE:**

The discovery of *Bacillus haimaensis* sp. nov. in the Haima cold seep of the South China Sea represents a significant advancement in our understanding of microbial adaptations to extreme marine environments. This novel species exhibits remarkable metabolic versatility and unique genomic features, providing insights into bacterial survival strategies in nutrient-variable, high-pressure deep-sea ecosystems. Comprehensive genomic analysis reveals distinctive biosynthetic gene clusters, suggesting untapped potential for discovering novel natural product. Furthermore, *B. haimaensis* exhibits promising capabilities for aromatic compound degradation, indicating potential applications in marine bioremediation. This work not only expands our knowledge of microbial diversity in understudied deep-sea habitats but also highlights the biotechnological promise of extremophiles. The adaptive mechanisms elucidated in *B. haimaensis*, particularly those related to sporulation and sulfate assimilation, contribute to our broader understanding of microbial ecology in cold seeps and may inform future research on climate change impacts on deep-sea ecosystems.

## INTRODUCTION

Deep-sea cold seeps are unique ecosystems characterized by the seepage of methane-rich fluids, supporting diverse microbial communities that play crucial roles in global biogeochemical cycles (1, 2). These extreme environments harbor microorganisms with specialized metabolic capabilities, serving as potential sources of novel bioactive compounds and biotechnological applications (3–5). Despite their ecological and biotechnological significance, our understanding of microbial diversity and adaptations in cold seeps, particularly in the South China Sea, remains limited (6, 7).

The genus *Bacillus*, comprising over 110 validly published species, is renowned for its metabolic diversity and endospore formation, enabling survival in extreme conditions (8, 9). *Bacillus* species have been isolated from various extreme habitats, including hydrothermal vents and hypersaline environments (10, 11). Their robust metabolic capabilities and stress resistance mechanisms make them promising candidates for biotechnological applications, including probiotics, enzyme production, and bioremediation (12–14).

Recent advancements in genomic technologies have revolutionized microbial taxonomy and functional characterization. Whole-genome sequencing and comparative genomics now provide deeper insights into bacterial adaptations and metabolic potential than traditional methods (15). These approaches are particularly valuable for understanding the unique features of extremophiles from underexplored environments like deep-sea cold seeps (16, 17).

The Haima cold seep, discovered in 2015 on the northwestern slope of the South China Sea, is one of only two active cold seep sites in this region (18). Despite its recent discovery, little is known about the microbial communities and their functional roles in this ecosystem. Exploring the microbial diversity of the Haima cold seep is crucial for understanding the ecological dynamics of these environments and for uncovering novel microbial resources with potential biotechnological applications (19, 20).

In this study, we report the isolation and characterization of a novel *Bacillus* species, designated *Bacillus haimaensis* sp. nov., from sediments of the Haima cold seep. Through a comprehensive approach combining phenotypic characterization, phylogenetic analysis, and comparative genomics, we provide insights into the adaptive strategies of this bacterium to its extreme habitat. Our findings not only expand our knowledge of microbial diversity in deep-sea cold seeps but also highlight the potential of *B. haimaensis* for applications in bioremediation and natural product discovery. This research contributes to the broader understanding of bacterial adaptations to extreme marine environments and offers a new model organism for studying life in deep-sea ecosystems.

## MATERIALS AND METHODS

### Sample collection and bacterial isolation

Sediment samples were collected from the Haima cold seep (16.73°N, 110.475°E) in the South China Sea at a depth of 1,350 m. Bacterial strains were isolated using a modified protocol (6). Briefly, 5 g of sediment was suspended in 40 mL sterile water, and 100 µL of a 100-fold diluted suspension was spread onto 2216E agar medium (Haibo, Qingdao, China). Cultures were incubated aerobically at 28°C for 1 week. Individual colonies were isolated and purified by repeated streaking.

### Strain identification and preservation

The 16S rRNA gene of each isolation was amplified using universal primers 27F and 1492R. PCR products were sequenced by Tsingke Biotechnology, China. Preliminary taxonomic identification was performed using the EzBioCloud online server (21). Based on these results, strain CSS-39^T^ was selected for further characterization.

### Phenotypic characterization

Cell morphology was examined using transmission electron microscopy (TEM) after negative staining with 1% (w/v) phosphotungstic acid (16). Growth characteristics under various conditions (temperature, salinity, pH) were evaluated in a 2216E liquid medium, with growth measured spectrophotometrically at 600 nm (OD_600_) (22). The base 2216E medium contained (per liter of deionized water) Bacto peptone (Difco) 5.0 g, yeast extract 1.0 g, ferric citrate 0.1 g, sodium chloride 19.45 g, magnesium chloride 8.8 g, sodium sulfate 3.24 g, calcium chloride 1.8 g, potassium chloride 0.55 g, sodium bicarbonate 0.16 g, potassium bromide 0.08 g, strontium chloride 34.0 mg, boric acid 22.0 mg, sodium silicate 4.0 mg, sodium fluoride 2.4 mg, ammonium nitrate 1.6 mg, and disodium hydrogen phosphate 8.0 mg. The medium was adjusted to pH 7.6 ± 0.2 and then autoclaved at 121°C for 15 min. For salt tolerance studies, we prepared a 2.1× concentrated base medium without NaCl and supplemented it with NaCl to achieve final concentrations ranging from 1% to 6% (w/v), corresponding to 0.21–1.34 g NaCl per 10 mL medium. pH tolerance was assessed using double-strength 2216E medium mixed with an equal volume of appropriate buffer solutions: citrate-phosphate buffer for pH 5–7 (0.2 M Na_2_HPO_4_: 0.1 M citric acid), Tris-HCl buffer for pH 8–9 (0.1 M HCl: 0.1 M Tris), and carbonate-hydroxide buffer for pH 10–11 (0.2 M NaOH: 0.05 M NaHCO_3_). Specific buffer ratios were used to achieve each pH value: pH 5.0 (10.30: 9.70), pH 6.0 (12.63: 7.37), pH 7.0 (16.47: 3.53), pH 8.0 (29.20: 50.00), pH 9.0 (5.70: 50.00), pH 10.0 (10.70: 50.00), and pH 11.0 (22.70: 50.00). All buffer solutions were diluted to 100 mL with deionized water, pH-adjusted if necessary, and autoclaved at 121°C for 20 min. Carbon source utilization was assessed using the Biolog GEN III microstation system (Biolog, USA) following the manufacturer’s instructions (23), focusing on environmentally relevant compounds. The selected carbon sources represent substrates commonly found in marine environments and allow direct comparison with previously characterized related species, particularly *Bacillus tianshenii* DSM 25879^T^.

### Genome sequencing, assembly, and annotation

Genomic DNA was extracted using a bacterial DNA kit (Omega, USA). Single-molecule real-time sequencing was performed by Novogene Technology Co., Ltd (Beijing, China). Sequencing reads were assembled into contigs using Canu v2.0 (24). Genome sequencing was performed using the PacBio Sequel II platform with a 10 kb insert library. Raw data generation yielded 1.03 Gb with an average read length of 15 kb, providing 264× genome coverage. Assembly using Canu v2.0 was performed with parameters: genomeSize = 3.2 m, correctedErrorRate = 0.045, minReadLength = 2,000, minOverlapLength = 500. Quality assessment using QUAST v5.0.2 (25) revealed a final assembly with one contig, N50 of 4.54 Mb, and genome completeness of 99.8% as assessed by BUSCO v5.2.2. The draft genome sequence has been deposited in GenBank under the accession number CP158164.

Genome sequence data were analyzed using a comprehensive bioinformatic pipeline. Gene prediction was performed using Prodigal v2.6.3 (26) with default parameters (minimum gene length: 90 bp, translation table: 11). Predicted protein-coding sequences were functionally annotated using DIAMOND v2.0.6 (e-value threshold: 1e-5, identity cutoff: ≥40%, query coverage: ≥70%) against the NCBI non-redundant (NR) database (27). Further functional annotation was carried out using the KEGG database via KofamKOALA (score threshold: system-defined adaptive score) (28) and the RAST server v2.0 (genetic code: 11, sequence type: DNA) (29, 30).

Genomic islands were identified using IslandViewer 4 (31), integrating four complementary approaches: IslandPath-DIMOB, SIGI-HMM, IslandPick, and Islander (minimum island size: 8 kb). Biosynthetic gene clusters were predicted using antiSMASH v7.0 (strict detection mode, minimum cluster size: 10 kb, minimum protein sequence identity: 30%) (32). A genome circle map was generated using the Proksee server with default visualization parameters (33).

### Phylogenetic and comparative genomic analyses

16S rRNA sequences of related strains were retrieved from the NCBI database. Sequence alignment was performed using Clustal W (34). A Neighbor-Joining phylogenetic tree based on 16S rRNA sequences was constructed using MEGA-X (35) and refined using iTOL v6 (36).

For comparative genomic analysis, whole-genome sequences of *B. tianshenii* DSM 25879^T^ (GenBank accession: GCA_016908565.1) and *B. tianshenii* SY-1-9 (GenBank accession: GCA_020177275.1) were retrieved from the NCBI genome database. These strains were selected based on their close phylogenetic relationship with CSS-39^T^ and their shared marine habitat origin, offering valuable insights into adaptive evolution in deep-sea environments. Average nucleotide identity (ANI) was calculated using the OrthoANI tool with a fragment size of 1,000 bp and a minimum alignment length of 700 bp. Species delineation threshold was set at ANI ≥ 95% (21). Digital DNA-DNA hybridization (dDDH) values were estimated using the Type (Strain) Genome Server via the Genome BLAST Distance Phylogeny (GBDP) method (formula d4, alignment length: ≥100 bp, similarity threshold: ≥30%). The species-level cutoff was set at dDDH ≥70%. The whole-genome sequence-based phylogenetic tree was constructed using the GBDP method (FastME algorithm with SPR postprocessing, 100 pseudo-bootstrap replicates) (37, 38). Comparative genomic analysis was performed using OrthoVenn3 (39) with the following parameters: e-value cutoff, 1e-5; inflation value, 1.5; minimum cluster size, 2; and protein overlap cutoff, 0.8. Orthologous clusters were identified based on reciprocal best hits from all-against-all protein sequence comparisons.

## RESULTS

### Morphological and growth characteristics

**Fig 1:**
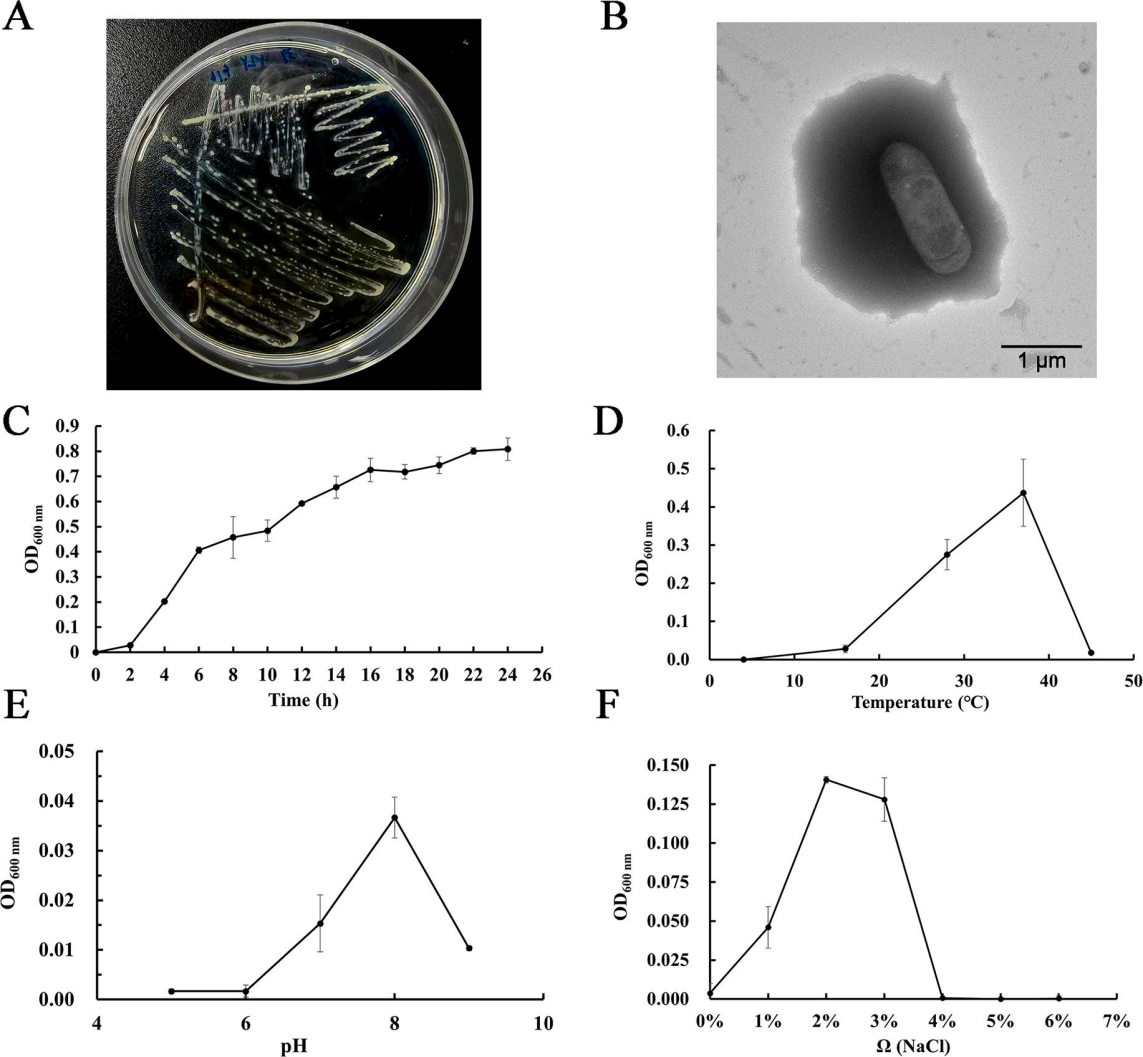
Morphological and physiological characteristics of *Bacillus* sp. CSS-39^T^. (**A**) Colony appearance of CSS-39^T^ on 2216E agar medium after 1 day of incubation. Colonies appear round, smooth, and light-yellow in color. (**B**) Transmission electron micrograph showing the cell of CSS-39^T^. The magnification is 8,000×. Bar: 500 nm. (**C**) Growth curve of CSS-39^T^ at 28°C. (**D**) Growth of CSS-39^T^ at different pH values. (**E**) Growth of CSS-39^T^ at different temperatures. (**F**) Growth of CSS-39^T^ under different salinities. Error bars in graphs C–F represent standard deviation from triplicate experiments. OD600nm indicates optical density measured at 600 nm wavelength, used as a proxy for bacterial growth.

Strain CSS-39^T^ was isolated from sediment collected in the Haima cold seep in the South China Sea. On 2216E agar medium, CSS-39^T^ formed round, smooth, light-yellow colonies after 24 h of incubation at 28°C (Fig. 1A). TEM analysis showed that cells of CSS-39^T^ were rod-shaped, measuring 1.5–2.3 μm in length and 0.4–0.7 μm in width, with no flagella or pili observable (Fig. 1B). CSS-39^T^ exhibited growth across a wide range of environmental conditions. The strain grew at temperatures between 4°C and 45°C, with optimal growth observed at 37°C (Fig. 1C). It tolerated pH values from 6 to 9, with optimal growth at pH 8 (Fig. 1D). The strain demonstrated halotolerance, growing in NaCl concentrations ranging from 0% to 4%, with optimal growth at 2% (Fig. 1E and F). These characteristics suggest adaptability to the variable conditions of deep-sea cold seeps, potentially contributing to the strain’s ecological success in this extreme environment.

### Physiological and biochemical characteristics

Biolog GEN III analysis revealed that CSS-39^T^ could utilize a diverse array of carbon sources (Table 1). The strain showed positive utilization of several carbohydrates including D-maltose, D-trehalose, D-cellobiose, sucrose, D-turanose, D-salicin, N-acetyl-D-glucosamine, α-D-glucose, D-mannose, and D-fructose. Among sugar alcohols, D-mannitol and myo-inositol were metabolized. The strain also utilized L-fucose and showed positive results for L-alanine, L-arginine, and L-glutamic acid utilization. Notably, CSS-39^T^ exhibited distinct metabolic capabilities compared to its closest relative, *B. tianshenii* DSM 25879^T^. While both strains could utilize D-maltose and α-D-glucose, CSS-39^T^ uniquely metabolized D-trehalose, D-cellobiose, sucrose, D-turanose, D-salicin, N-acetyl-D-glucosamine, D-mannose, D-fructose, L-fucose, D-mannitol, and myo-inositol. CSS-39^T^’s ability to metabolize a wider range of sugars and amino acids suggests a more versatile metabolic capability, which may contribute to its adaptation to the nutrient-variable environment of deep-sea cold seeps. This metabolic flexibility could provide a competitive advantage in colonizing and thriving in such extreme habitats.

**TABLE 1:** Comparison of carbon source utilization between CSS-39^T^ and its closely related strain *B. tianshenii* DSM 25879^T^^*a*^

Carbon source utilization	*Bacillus* sp. CSS-39^T^	*B. tianshenii* DSM 25879^T^
D-Maltose	+	+
D-Trehalose	+	−
D-Cellobiose	+	−
Gentiobiose	−	−
Sucrose	+	−
D-Turanose	+	−
D-Raffinose	−	−
α-D-Lactose	−	−
D-Melibiose	−	−
D-Salicin	+	−
N-Acetyl-D-Glucosamine	+	−
α-D-Glucose	+	+
D-Mannose	+	−
D-Fructose	+	−
D-Galactose	−	−
D-Fucose	−	−
L-Fucose	+	−
L-Rhamnose	−	−
D-Sorbitol	−	−
D-Mannitol	+	−
D-Arabitol	−	−
myo-Inositol	+	−
Glycerol	−	+
Gelatin	−	+
L-Alanine	+	+
L-Arginine	+	−
L-Glutamic Acid	+	+
L-Histidine	−	+
Tween 40	−	+

^*a*^ Data of *B. tianshenii* DSM 25879^T^ are from Jiang et al. (22). + indicates positive utilization/metabolism of the substrate, and − indicates inability to utilize the substrate.

### Phylogenetic analysis and taxonomic position

**Fig 2:**
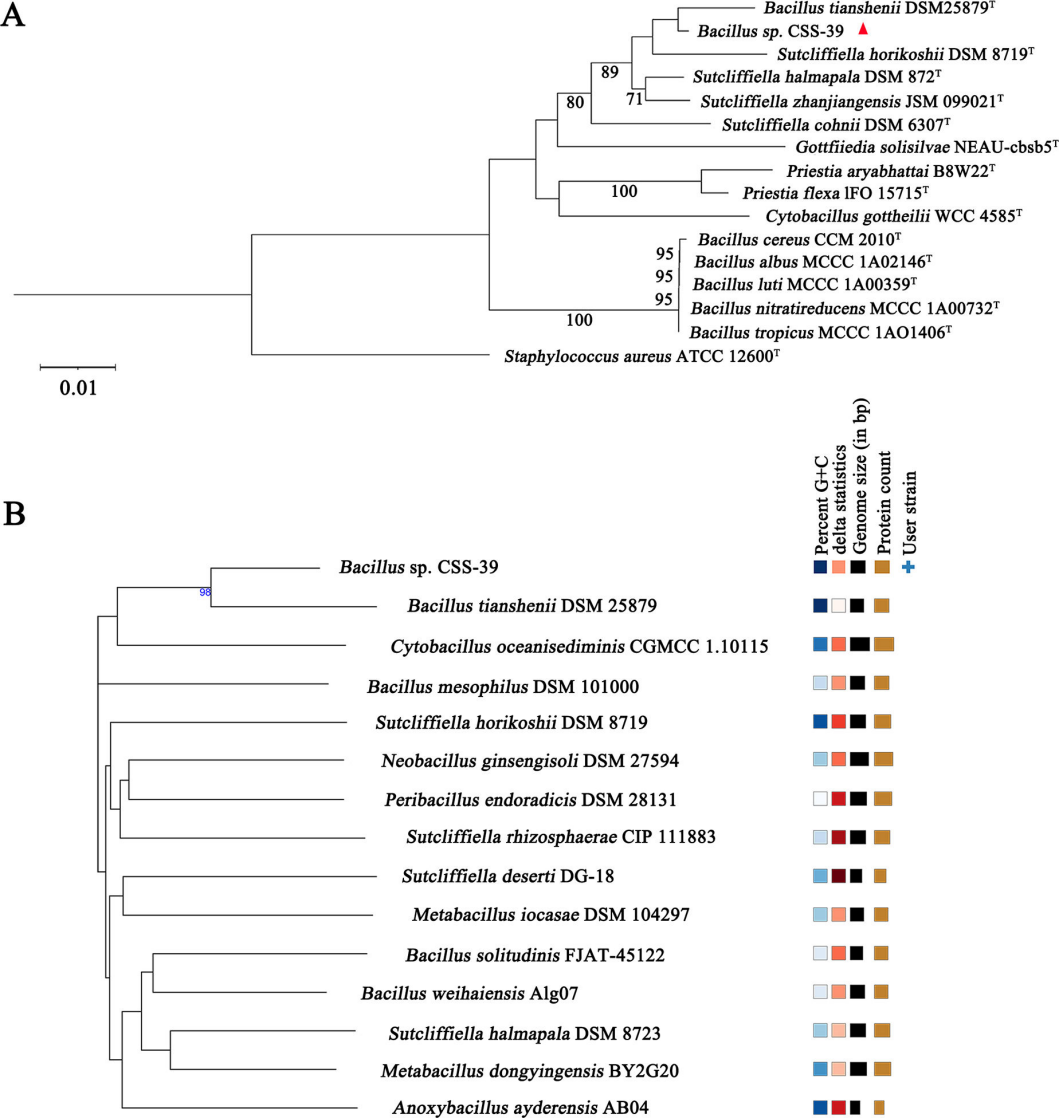
Phylogenetic analysis of *Bacillus* sp. CSS-39^T^. (**A**) Neighbor-joining phylogenetic tree based on 16S rRNA gene sequences. The tree was constructed with 1,000 bootstrap replicates. Bootstrap values (%) are shown at branch nodes. *Staphylococcus aureus* ATCC 12600^T^ was used as an outgroup. The scale bar represents 0.01 substitutions per nucleotide position. (**B**) Phylogenetic tree of whole-genome sequences constructed by GBDP method. Clade lengths were scaled according to the GBDP distance formula *d_5_*.

Phylogenetic analysis based on 16S rRNA gene sequences positioned CSS-39^T^ within the genus *Bacillus*, with its closest relative being *B. tianshenii* DSM 25879^T^ (99.01% sequence identity) (Fig. 2A). Whole-genome-based phylogenetic analysis using the GBDP method provided higher resolution, clearly delineating CSS-39^T^ as a distinct lineage within the *Bacillus* genus (Fig. 2B). This discrepancy between 16S rRNA-based and whole-genome-based phylogenetic analyses underscores the importance of using comprehensive genomic approaches for accurate taxonomic classification, particularly in closely related bacterial species. The distinct phylogenetic position of CSS-39^T^, combined with its unique morphological and metabolic characteristics, strongly supports its classification as a novel species within the *Bacillus* genus.

### Genomic relatedness and species delineation

To further establish the taxonomic position of strain CSS-39^T^, we calculated genomic relatedness indices with its closest phylogenetic neighbors (Tables 2 and 3). Average nucleotide identity (OrthoANI) values between CSS-39^T^ and its closest relatives, *B. tianshenii* DSM 25879^T^ and *B. tianshenii* SY-1-9, were 87.78% and 87.56%, respectively (Table 2). These values fall well below the 95%–96% threshold typically used for species delineation, providing strong evidence for CSS-39^T^’s distinct species status.

**TABLE 2:** OrthoANI values between CSS-39^T^ and strains of *B. tianshenii*

	*Bacillus tianshenii* DSM 25879^T^ (A) & *Bacillus *sp. CSS-39^T^ (B)	*Bacillus tianshenii* SY-1-9 (A) & *Bacillus* sp. CSS-39^T^ (B)
OrthoANIu value (%)	87.78	87.56
Genome A length (bp)	4,201,380	4,230,960
Genome B length (bp)	4,544,100	4,544,100
Average aligned length (bp)	2,434,305	2,374,840
Genome A coverage (%)	57.94	56.13
Genome B coverage (%)	53.57	52.26

**TABLE 3:** dDDH values with confidence interval (CI) between CSS-39^T^ and related strains^*a*^

Query	Subject	*d*_0_	CI *d*_0_	*d*_4_	CI *d*_4_	*d*_6_	CI *d*_6_	Diff. G+C percent
CSS-39^T^	*Bacillus tianshenii* DSM 25879^T^	70.2	[66.3–73.9]	**34.0**	[31.6–36.5]	60.7	[57.5–63.9]	0.76
CSS-39^T^	*Sutcliffiella halmapala* DSM 8723^T^	20.8	[17.5–24.4]	**20.9**	[18.6–23.3]	19.9	[17.2–22.9]	4.81

^*a*^ *d*_0_, *d*_4_, and *d*_6_ represent different dDDH calculation methods, among which the result of *d*_4_ is not related to genome length and has higher recognition. The bold values (34.0 and 20.9) in the *d*_4_ column are emphasized because they represent the most reliable dDDH calculation method among the three approaches (*d*_0_, *d*_4_, and *d*_6_). The *d*_4_ method is specifically highlighted as it is independent of genome length and provides superior discriminatory power for species delineation, making these values particularly crucial for taxonomic classification.

The dDDH analysis revealed values of 34.0% [31.6%–36.5%] between CSS-39^T^ and *B. tianshenii* DSM 25879^T^, with a G+C content difference of 0.76% (Table 3). This dDDH value falls well below the 70% threshold recommended for species delineation, supporting the classification of CSS-39^T^ as a distinct species. The comparison with *Sutcliffiella halmapala* DSM 8723^T^, a recently reclassified member of the family *Bacillaceae* that shares similar environmental adaptations, showed an even lower dDDH value of 20.9% [18.6%–23.3%] and a larger G+C content difference of 4.81%. This comparison was included because *S. halmapala* represents a phylogenetically related but distinct lineage within the *Bacillaceae* that has recently undergone taxonomic revision, helping to establish clear boundaries between genera in this diverse family.

The consistently low dDDH values across different calculation methods (d0, d4, and d6) provide robust evidence for the novel species status of strain CSS-39^T^. Notably, the d4 calculation method, which is independent of genome length and provides higher precision for species delineation, also strongly supports this classification. These genomic indices, together with the phylogenetic analysis and distinctive phenotypic characteristics, provide comprehensive evidence for classifying strain CSS-39^T^ as a novel species within the genus *Bacillus*, for which we propose the name *B. haimaensis* sp. nov.

### Genome features and functional annotation

**Fig 3:**
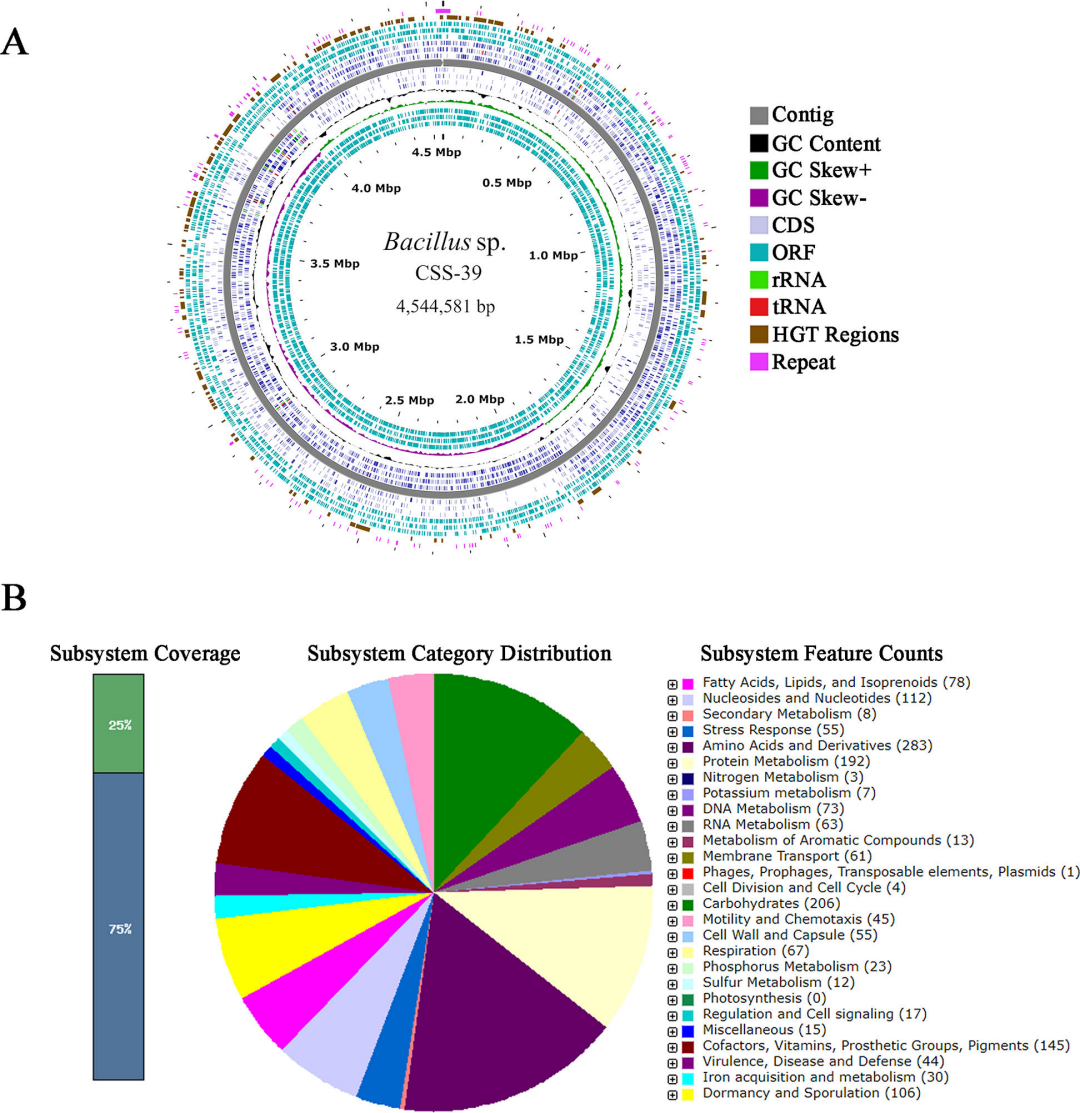
General genome characterizations of CSS-39^T^. (**A**) Genomic circle map of CSS-39^T^. The outermost circle represents the scale in Mbp. Moving inwards, the subsequent circles depict CDS on the forward strand (light blue), ORF on the reverse strand (dark blue), tRNA genes (red), rRNA genes (green), GC content (black), and GC skew (purple/green). (**B**) Functional categorization of the CSS-39^T^ genome based on RAST annotation. The pie chart illustrates the distribution of subsystem categories, with the size of each slice proportional to the number of features in that category. The bar graph on the left shows the overall subsystem coverage.

The complete genome of CSS-39^T^ consists of a single circular chromosome of 4,544,581 bp with a GC content of 42.4% (Fig. 3A). Genome annotation identified 4,546 predicted open reading frames (ORFs), of which 4,477 (98.48%) were annotated in the NCBI NR database. RAST annotation revealed 27 rRNA sequences, 83 tRNA sequences, and 4,813 coding sequences (CDS). Functional categorization highlighted significant representations in amino acids and derivatives (283 subsystems), carbohydrates (206 subsystems), and protein metabolism (192 subsystems) (Fig. 3B).

**Fig 4:**
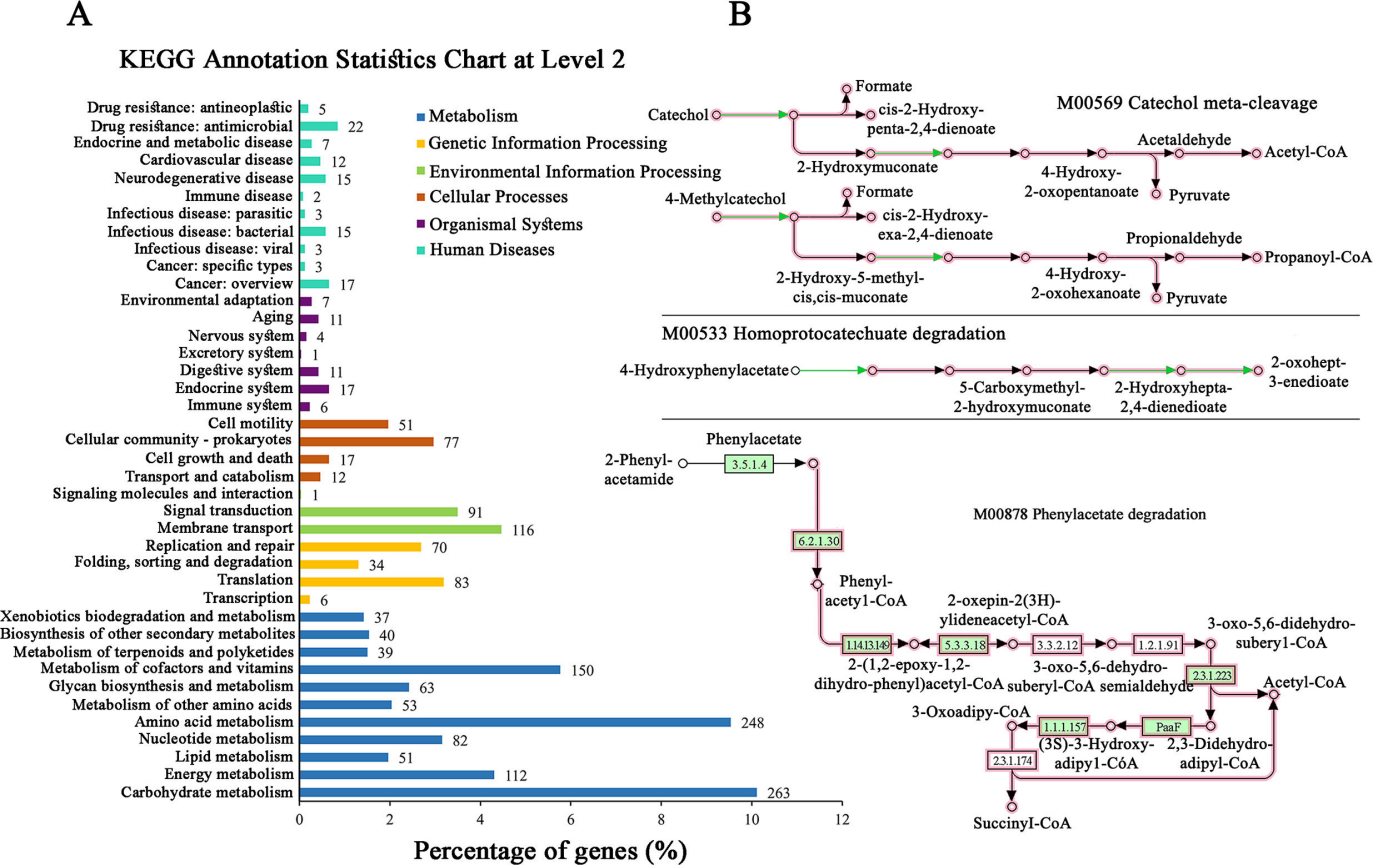
KEGG pathway analysis and aromatic compound degradation capabilities of *Bacillus* sp. CSS-39^T^. (**A**) KEGG Annotation Statistics Chart at Level 2. This chart illustrates the functional distribution of genes in CSS-39^T^ according to KEGG pathways. The *x*-axis represents the percentage of genes in each level 2 category relative to all KEGG-annotated genes. The *y*-axis lists the level 2 KEGG pathway classifications. Numbers at the end of each bar indicate the absolute count of genes in that category. (**B**) Degradation pathways of aromatic compounds in which CSS-39^T^ is involved. The green annotation is the product of genes that can be annotated in this strain, and the pink annotation is the KEGG module, which is a collection of manually defined functional units. The module number and name have been marked in the figure.

KEGG pathway analysis showed a predominance of genes involved in carbohydrate metabolism (263 genes), amino acid metabolism (248 genes), and metabolism of cofactors and vitamins (150 genes) (Fig. 4A). Notably, we identified genes encoding key enzymes for aromatic compound degradation, including catechol 2,3-dioxygenase (EC: 1.13.11.2) and phenylacetyl-CoA ligase (EC: 6.2.1.30) (Fig. 4B). Similar aromatic degradation pathways have been experimentally validated in other deep-sea bacteria, such as *Abyssisolibacter* sp. M8S5 from cold seep sediments (22). These genomic features suggest a metabolically versatile organism adapted to the heterogeneous nutrient landscape of cold seep environments. The presence of aromatic compound degradation pathways indicates potential applications in marine bioremediation, highlighting the biotechnological significance of this novel species.

### Genomic islands and secondary metabolite biosynthesis

**Fig 5:**
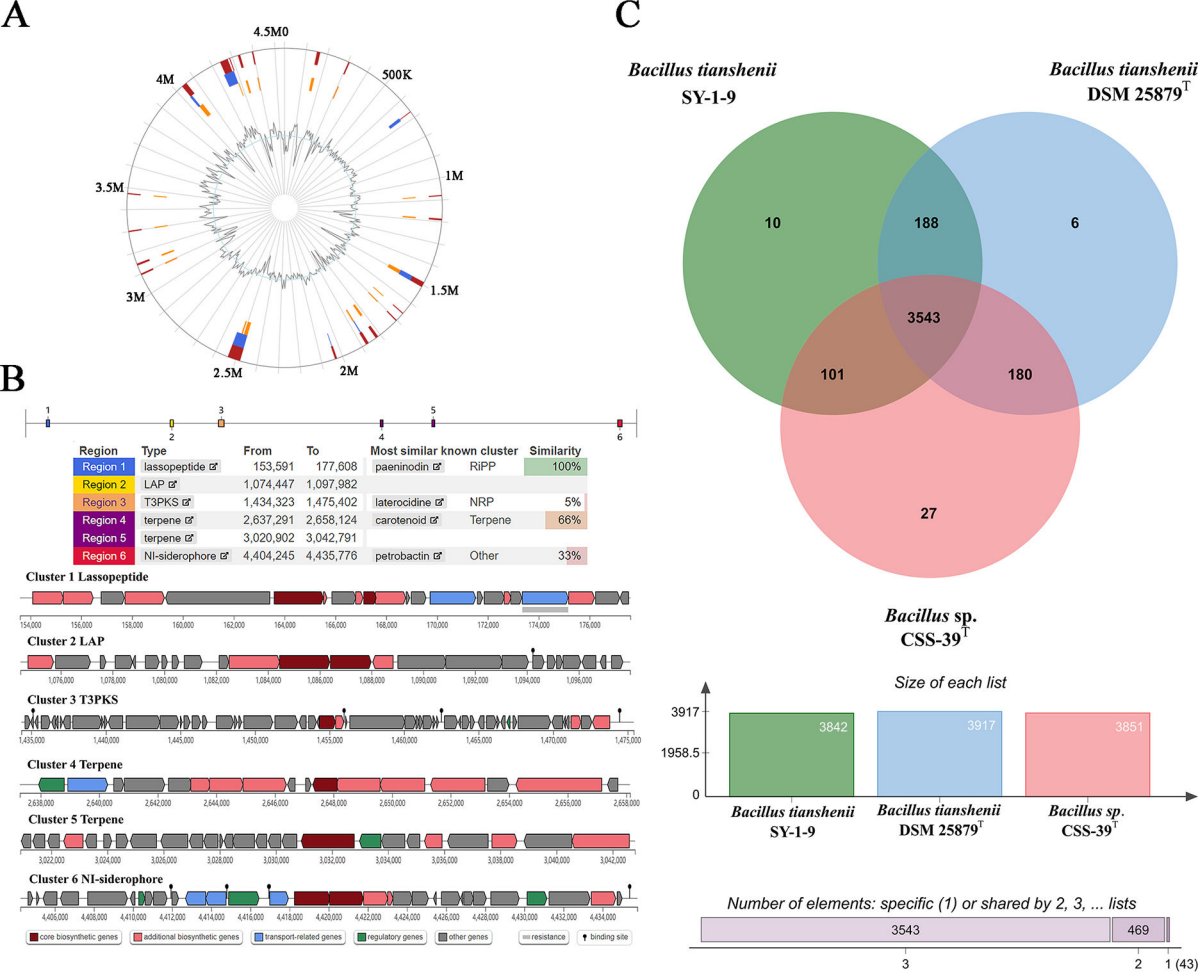
Genomic features and comparative analysis of *Bacillus* sp. CSS-39^T^. (**A**) Genomic islands (GIs) prediction in CSS-39^T^. The circular representation shows the distribution of predicted GIs across the genome. Orange segments indicate GIs predicted by the SIGI-HMM method, blue segments represent GIs identified by the IslandPath-DIMOB method, and red segments show the consensus predictions from multiple methods. The inner grey line represents GC content variation. (**B**) Biosynthetic gene clusters (BGCs) predicted in CSS-39^T^. Six distinct BGCs are illustrated, including lassopeptide, linear azole-containing peptide (LAP), type III polyketide synthase (T3PKS), terpene, and non-ribosomal peptide synthetase (NRPS) clusters. Each cluster is represented by a schematic showing gene organization, with different colors indicating various functional components. (**C**) Orthologous gene cluster analysis comparing CSS-39T with closely related species. The Venn diagram illustrates the distribution of shared and unique gene clusters among CSS-39^T^ (pink), *B. tianshenii* SY-1-9 (green), and *B. tianshenii* DSM 25879^T^ (blue). Numbers in each section represent the count of gene clusters. The accompanying histogram quantifies the specific and shared gene clusters across the three genomes.

Analysis using IslandViewer 4 identified 21 distinct genomic islands in CSS-39^T^ (Table S1), ranging in size from 3,318 bp to 59,622 bp. The largest genomic island (coordinates 4239473–4277188) contains 41 genes, including the complete ATP synthase operon and NADH-ubiquinone oxidoreductase complex. Of the 162 total genes within these islands, 89 (54.9%) encode hypothetical proteins, while the remaining genes encode various functional proteins including metabolic enzymes (e.g., thymidylate synthase, UDP-glucose 4-epimerase), transport-related proteins (e.g., chromate transporters, sulfate permeases), transcriptional regulators, and four spore formation/germination-related proteins (including spore germination proteins and stage II sporulation protein D) (Table S1; Fig. 5A). Several islands contain gene clusters for polysaccharide biosynthesis and modification. Notably, our comprehensive analysis revealed that these genomic islands contain multiple nutrient acquisition-related genes that are particularly relevant to cold seep environments. The transport-related genes include ABC transporters and ion channels for various substrates, chromate transporters for metal ion transport, sulfate permeases for sulfur compound uptake, and substrate-specific permeases for selective nutrient absorption. Moreover, we identified several metabolic adaptation genes, including UDP-glucose 4-epimerase for carbohydrate metabolism, genes involved in amino acid transport and metabolism, and various substrate-specific metabolic enzymes. These findings suggest the acquisition of metabolic capabilities through horizontal gene transfer that may enhance adaptation to the nutrient-variable cold seep environment.

AntiSMASH analysis revealed six biosynthetic gene clusters (BGCs) (Fig. 5B): one lassopeptide cluster showing 100% similarity to the paeninodin biosynthesis cluster from *B. paenibacillus* OSY-SE (MIBiG BGC0001874), one type III PKS cluster (38% similarity to alkylresorcinol synthase cluster), two NRPS clusters (22% and 15% similarity to known clusters), one terpene cluster (45% similarity to carotenoid biosynthesis cluster), and one LAP cluster showing no significant similarity to known clusters. The presence of these unique genomic islands and diverse BGCs suggests that *B. haimaensis* sp. nov. has evolved specific adaptations to its extreme habitat. Furthermore, the low similarity of most BGCs to known clusters indicates potential for novel secondary metabolite production, which could have significant implications for natural product discovery and pharmaceutical research.

### Comparative genomic analysis

Orthologous gene cluster analysis among CSS-39^T^, *B. tianshenii* DSM 25879^T^, and *B. tianshenii* SY-1-9 identified 3,543 core orthologous gene clusters, primarily related to metabolic processes such as nitrogen compound metabolism, aromatic compound metabolism, and heterocyclic metabolism (Fig. 5C). Importantly, CSS-39^T^ possessed 27 unique gene clusters (Table S2), including those related to spore germination and sulfate assimilation. Similar spore formation capabilities have been experimentally demonstrated in other deep-sea *Bacillus* species as a survival mechanism under extreme conditions (9). The presence of sulfate assimilation genes aligns with previous studies showing active sulfur metabolism in cold seep bacteria (6). This genomic distinctiveness suggests enhanced metabolic efficiency and potential adaptation to the extreme cold seep environment. The unique gene clusters, particularly those involved in spore germination and sulfate assimilation, may provide *B. haimaensis* sp. nov. with competitive advantages in colonizing and surviving in its challenging habitat. These findings support novel species status of CSS-39^T^ and provide insights into the genomic basis of bacterial adaptation to deep-sea cold seeps.

## DISCUSSION

In this study, we isolated and characterized a novel *Bacillus* species, *B. haimaensis* sp. nov., from the Haima cold seep in the South China Sea. Our comprehensive analysis, combining phenotypic characterization, phylogenetic analysis, and comparative genomics, robustly supports its classification as a new species and offers insights into its adaptations to the cold seep environment.

The taxonomic status of bacteria has traditionally been determined using a combination of 16S rRNA gene sequence analysis, phenotypic characterization, and DNA-DNA hybridization experiments. However, the advent of whole-genome sequencing has revolutionized bacterial taxonomy, offering more precise methods for species delineation (15). In our study, while the 16S rRNA gene sequence similarity between *B. haimaensis* sp. nov. and its closest relative, *B. tianshenii* DSM 25879^T^, was 99.01%, exceeding the traditional 97% species boundary, genomic indices provided clear evidence of species-level differentiation. The OrthoANI and dDDH values between *B. haimaensis* sp. nov. and *B. tianshenii* DSM 25879^T^ were 87.78% and 34.0%, respectively, well below the established thresholds for species delineation (95%–96% for ANI and 70% for dDDH) (38, 40). These findings support the growing consensus that whole-genome-based methods offer superior resolution in bacterial taxonomy, particularly when 16S rRNA gene sequences show high similarity (41). Our results align with recent proposals to raise the 16S rRNA gene sequence identity threshold to 98.7% for preliminary species determination, with OGRI calculations recommended for final taxonomic decisions (15).

The metabolic profile of *B. haimaensis* sp. nov. reveals a remarkable versatility in carbon source utilization, surpassing that of its closest relative, *B. tianshenii* DSM 25879^T^. This enhanced metabolic capability, particularly in the utilization of various sugars and amino acids, likely represents an adaptation to the heterogeneous and potentially nutrient-limited environment of deep-sea cold seeps (23, 42). It is worth noting that *B. tianshenii* DSM 25879^T^, originally isolated from marine sediments at a depth of 652 m (43), shows a more limited carbon utilization profile. This difference suggests that *B. haimaensis* sp. nov. may have evolved additional metabolic capabilities to thrive in the more extreme cold seep environment at 1,350 m depth. The ability to metabolize a wide range of carbon sources is a common feature among heterotrophic bacteria isolated from cold seep sediments, suggesting its importance in adapting to these unique habitats (22).

Genomic analysis revealed that *B. haimaensis* sp. nov. possesses key genes encoding enzymes involved in aromatic compound degradation pathways, notably catechol 2,3-dioxygenase and phenylacetyl-CoA ligase. The presence of these enzymes is particularly relevant in cold seep environments, which are characterized by natural hydrocarbon seepage and complex organic matter (44). Recent metagenomic studies have demonstrated that cold seep microbial communities harbor diverse metabolic capabilities (22), with many bacteria showing specific adaptations for hydrocarbon degradation (45). Of particular ecological significance is the strain’s genomic potential for aromatic compound degradation, which suggests possible applications in marine bioremediation. However, experimental validation is needed to determine the actual substrate range and degradation capabilities of *B. haimaensis* sp. nov. under deep-sea conditions, especially regarding both naturally occurring and anthropogenic aromatic compounds. This is particularly important given that cold seeps represent dynamic environments with varying concentrations of both biogenic and thermogenic hydrocarbons. The genome also encodes pathways for sulfate assimilation, consistent with adaptation to the sulfur-rich conditions typical of cold seep environments (6). This combination of metabolic capabilities—aromatic compound degradation and sulfur metabolism—suggests that *B. haimaensis* sp. nov. is well-adapted to the geochemical conditions of cold seep ecosystems.

Comparative analysis with other cold seep bacteria reveals interesting parallels in adaptation strategies. For instance, *Campylobacterota* isolated from the Formosa cold seep show specialized metabolic capabilities for nitrogen and sulfur cycling (46), while *Pseudomonas marinensis* isolated from South China Sea cold seeps exhibits unique cold-active lipases (4). Like *B. haimaensis* sp. nov., these bacteria demonstrate metabolic versatility and specific adaptations to the cold seep environment. Recent metagenomic studies of South China Sea cold seeps have revealed diverse bacterial communities dominated by Proteobacteria, Bacteroidota, and Firmicutes (44), suggesting complex ecological networks in which *B. haimaensis* sp. nov. likely plays an important role.

The adaptability of cold seep bacteria varies significantly across species. For example, while some *Sulfurovum* species are strictly anaerobic chemolithotrophs (46), *B. haimaensis* sp. nov. shows remarkable metabolic flexibility as a facultative anaerobe. This metabolic versatility may represent a different evolutionary strategy for surviving in the dynamic cold seep environment. Similarly, while *Abyssisolibacter* species from cold seeps show specialized adaptations for carbohydrate metabolism (22), *B. haimaensis* sp. nov. exhibits broader substrate utilization capabilities.

Comparative genomic analysis revealed 27 unique gene clusters (Table S2) in *B. haimaensis* sp. nov., providing insights into its adaptation to the cold seep environment. The presence of three spore germination-related gene clusters (GO:0009847) suggests enhanced sporulation capabilities, which likely enable survival under fluctuating environmental conditions characteristic of cold seeps (20). The identification of two sulfate assimilation clusters (GO:0000103) indicates adaptation to sulfur-rich conditions, while multiple stress-response genes, including those for oxidative stress response (GO:0006979), toxic substance response (GO:0009636), and peroxiredoxin activity (GO:0051920), likely contribute to environmental resilience. The genome also encodes diverse metabolic functions, including metal ion binding (GO:0046872), serine-type endopeptidase activity (GO:0004252), and glycosyl transferase activity (GO:0016757), suggesting metabolic versatility in resource utilization. The identification of genomic islands containing these adaptive traits indicates their likely acquisition through horizontal gene transfer, a mechanism known to facilitate bacterial niche adaptation (31). This genomic plasticity appears to have enabled successful colonization of the cold seep habitat, where fluctuating conditions and high sulfur content present significant challenges to microbial survival. The combination of these genomic features provides a molecular foundation for understanding how *B. haimaensis* sp. nov. has adapted to thrive in the demanding cold seep environment.

The discovery of six BGCs in *B. haimaensis* sp. nov., including a cluster with 100% similarity to a known paeninodin biosynthesis cluster, highlights its potential for secondary metabolite production. The paeninodin cluster is particularly interesting due to its unique phosphorylation modification step in biosynthesis, which has been experimentally characterized in related *Bacillus* species and shown to contribute to novel bioactive properties (47, 48). Marine *Bacillus* species are known to be prolific producers of bioactive compounds, including antimicrobial and anticancer agents (49, 50). The unique environmental pressures of cold seeps may have driven the evolution of novel biosynthetic pathways in *B. haimaensis* sp. nov., making it a promising candidate for natural product discovery. The identification of a terpene biosynthesis cluster is particularly intriguing, given the growing interest in marine-derived terpenes for drug development (51, 52). The low similarity of several BGCs to known clusters suggests the potential for discovery of novel secondary metabolites, further emphasizing the biotechnological promise of *B. haimaensis* sp. nov.

The isolation of *B. haimaensis* sp. nov. from the Haima cold seep contributes to our understanding of microbial diversity in these understudied ecosystems. Cold seeps are known to harbor diverse microbial communities that play crucial roles in biogeochemical cycles (1, 2). The metabolic versatility and unique genomic features of *B. haimaensis* sp. nov. provide insights into the strategies employed by bacteria to thrive in these extreme environments.

Several limitations of our study should be acknowledged. While we have characterized the genomic and phenotypic features of *B. haimaensis* sp. nov., its ecological interactions within the cold seep community remain largely unknown. Although we identified several biosynthetic gene clusters, their actual products and ecological functions require experimental verification. Additionally, the regulatory mechanisms controlling the strain’s metabolic versatility and stress responses need further investigation.

Future research should address these limitations through multiple approaches. Meta-transcriptomic studies will be essential to understand the active roles of *B. haimaensis* sp. nov. *in situ*, while investigation of potential syntrophic relationships with other cold seep microorganisms will reveal its ecological interactions. Priority should be given to experimental characterization of the identified biosynthetic gene clusters and detailed analysis of stress response mechanisms under high-pressure and low-temperature conditions. Furthermore, exploration of potential biotechnological applications, particularly in bioremediation and natural product discovery, warrants investigation.

In conclusion, the discovery and characterization of *B. haimaensis* sp. nov. expand our knowledge of microbial diversity in deep-sea cold seeps and provide a new model organism for studying bacterial adaptations to extreme marine environments. Its unique metabolic and genomic features not only enhance our understanding of bacterial adaptation to cold seeps but also offer promising avenues for biotechnological applications.

### Conclusion

This study introduces *Bacillus haimaensis* sp. nov., a novel bacterial species isolated from the Haima cold seep in the South China Sea, representing a significant addition to our understanding of microbial diversity in extreme marine environments. Our multifaceted approach, combining phenotypic characterization, phylogenetic analysis, and comparative genomics, provides robust evidence for its classification as a novel species within the genus *Bacillus*. The genomic and phenotypic features of *B. haimaensis* sp. nov. reveal remarkable adaptations to the cold seep environment, including a versatile metabolic profile and unique gene clusters related to spore germination and sulfate assimilation. Of particular interest is its biosynthetic potential, evidenced by six biosynthetic gene clusters, some with low similarity to known clusters, opening new avenues for natural product discovery. Furthermore, the strain’s capability for aromatic compound degradation suggests potential applications in marine bioremediation. This study expands our knowledge of deep-sea microbial ecology and provides a new model organism for investigating bacterial adaptations to extreme conditions, underscoring the importance of exploring deep-sea environments as reservoirs of microbial diversity and functional novelty.

## Data Availability

The authors confirm that the data supporting the findings of this study are available within the article and its supplemental material. The genome information of Bacillus haimaensis CSS-39^T^ has been uploaded to NCBI under accession number CP158164. Strain CSS-39^T^ has been deposited in the China Center for Type Culture Collection (CCTCC) under accession number CCTCC AB 2025078 and in the Guangdong Microbial Culture Collection Center (GDMCC) under accession number GDMCC 1.5655.

## SUPPLEMENTAL MATERIAL

Table S1 aem.02456-24-s0001.xlsx Genomic islands and associated genes in *B. haimaensis* CSS-39^T^.

Table S2 aem.02456-24-s0002.xlsx Gene cluster analysis and Gene Ontology (GO) annotations of unique genes in *B. haimaensis* sp. nov.

ASM does not own the copyrights to Supplemental Material that may be linked to, or accessed through, an article. The authors have granted ASM a non-exclusive, world-wide license to publish the Supplemental Material files. Please contact the corresponding author directly for reuse.
