# Correction for Wang et al., “*Bacillus haimaensis* sp. nov.: a novel cold seep-adapted bacterium with unique biosynthetic potential”

**DOI:** 10.1128/aem.01157-26

**Published:** 2026-08-19

**Authors:** Yuanyuan Wang, Luyi Yang, Wenbo Wu, Zhengqi Feng, Jian He, Changjun Guo, Jianguo He

## AUTHOR CORRECTION

Volume 91, no. 5, e02456-24, 2025, https://doi.org/10.1128/aem.02456-24. During a post-publication review of our genome annotation results and related summary descriptions, we identified three locations where the published text did not accurately reflect the original annotation outputs and summary calculations. Some cluster types and similarity values were misdescribed.

Results, 1st paragraph of “Genomic islands and secondary metabolite biosynthesis” section: “Of the 162 total genes within these islands, 89 (54.9%) encode hypothetical proteins” should read “Of the 324 total genes within these islands, 166 (51.23%) encode hypothetical proteins.”

Results, 2nd paragraph of “Genomic islands and secondary metabolite biosynthesis” section: “one lassopeptide cluster showing 100% similarity to the paeninodin biosynthesis cluster from *B. paenibacillus* OSY-SE (MIBiG BGC0001874), one type III PKS cluster (38% similarity to alkylresorcinol synthase cluster), two NRPS clusters (22% and 15% similarity to known clusters), one terpene cluster (45% similarity to carotenoid biosynthesis cluster), and one LAP cluster showing no significant similarity to known clusters” should read “one lassopeptide cluster showing 100% similarity to the paeninodin biosynthesis cluster, one LAP cluster with no significant similarity to known clusters, one T3PKS cluster showing 5% similarity to the laterocidine biosynthesis cluster, one terpene cluster showing 66% similarity to the carotenoid biosynthesis cluster, one terpene cluster with no significant similarity to known clusters, and one NI-siderophore cluster showing 33% similarity to the petrobactin biosynthesis cluster.” Figure 5B correctly showed all six antiSMASH-predicted BGCs, including the second terpene cluster and the NI-siderophore cluster; these two clusters were inadvertently omitted from the text description.

Results, “Comparative genomic analysis” section: “CSS-39^T^ possessed 27 unique gene clusters” should read “CSS-39^T^ possessed 26 unique gene clusters.”

These corrections do not affect the overall conclusions of the article.

